# Development of thermoplastic polyurethane/polyaniline-doped membranes for the separation of glycine through electrodialysis

**DOI:** 10.3906/kim-1907-2

**Published:** 2020-02-11

**Authors:** Khurram ., Asif ALI QAISER, Rashid SALEEM, Naveed SHAHZAD ALI, Shahbaz NAZIR, Tousif HUSSAIN, Abdul GHAFFAR

**Affiliations:** 1 Department of Chemistry, University of Engineering and Technology, Lahore Pakistan; 2 Department of Polymer and Process Engineering, University of Engineering and Technology Lahore Pakistan; 3 Department of Chemistry, Government College University, Katchery Road, Lahore Pakistan; 4 Department of Chemistry, Punjab University, Lahore Pakistan; 5 Department of Center for Advanced Studies in Physics, Government College University, Lahore Pakistan

**Keywords:** Thermoplastic polyurethane, sulfonic acid dopants, electrodialysis, polyaniline, glycine

## Abstract

Recently, membrane-based separation processes, particularly electrodialysis, have attracted attention for the separation and purification of organic and amino acids from animal feedstock waste. In this study, cation exchange membranes were synthesized by making a composite of thermoplastic polyurethane and polyaniline (PANI) via the doping of various aromatic sulfonic acids, such as β -naphthol sulfonic acid and phenol sulfonic acid. The PANI was prepared using a standard method, which was further used in the composite blending at varying concentrations of 10%–20%. The impact of the concentration of PANI and the nature of the dopant on the membrane characteristics were comparatively studied. The membranes were analyzed by electric conductivity, water swelling, morphological studies (SEM), and thermogravimetric analysis. The membranes were used for the separation of glycine hydrochloride via electrodialysis.

## 1. Introduction

Intrinsically conducting polymers (ICPs) are auspicious materials for many applications. Polyaniline (PANI) is a conducting polymer that has been widely studied due to its environmental stability, low cost, and ease of synthesis. PANI can be synthesized by chemical or electrochemical methods. In either method, the conjugated monomer is polymerized and charge carriers are created using certain dopants. Here, the presence of the dopant is very important, because the charge carriers and conductivity of the polymer can be affected by the degree and type of dopant. Unfortunately, commercial applications of PANI are limited due to its poor processability and mechanical properties. To resolve these issues, various solutions have been investigated [1,2].

PANI exists in a various controllable oxidation states. One of which is as emeraldine salt, which is highly conductible. Emeraldine salt possesses an equal number of amine and imine sites in its molecular chain. The protonation of imine site converts it into bipolar emeraldine salt, and then by further rearrangement of the bipolar emeraldine salt, delocalized polaron lattice (polysemiquinone radical) salt is obtained [3,4]. Switchability of PANI can be seen by adding acids or bases that convert it into protonated and deprotonated forms. This shows that the dependence of the reaction and polymer state is based on the pH of the solution. At a pH greater than 4, the electroactivity of PANI is lost because the conductive form of PANI (emeraldine salt) does not exist [5]. No doubt, PANI possesses fantastic properties and that is why it is used in electrodialysis, nanofiltration, pervaporation, and fuel cells [6–8]. Instead of remarkable properties, pristine PANI exhibits poor mechanical properties, which are due to the chemical nature of PANI; it is intractable and brittle. This property results in fracturing of the membrane when even minimal stress is applied to it [9].

Considerable attention has been given to avoiding such limitations by blending PANI with other polymers. However, PANI is often immiscible with other polymers because the phase separation hinders it forming uniform material. Such problems may be subdued by using a suitable compatibilizer, such as an ionic or block polymer [10,11]. High compatibility of these blends results in less phase separation with higher mechanical and electrical properties. Thermoplastic polyurethane (TPU) is a versatile thermoplastic with elastomeric properties. It consists of 2-phase morphology, one of which is soft and is characterized by either polyesters or polyethers, while the other is hard and is characterized by aromatic diisocyanate with short-chain diol. Hence, the soft phase is reinforced and condensed with the hard one. Due to its extra ordinary physical properties, abrasion, chemical resistance, and ease of processability, keen interest to has developed to prepare its membranes with a conducting polymer like PANI.

The purpose of this research was to enhance the mechanical properties and processability of PANI by blending it with TPU, and then use it in electrodialysis as cation exchange membrane for the separation of compounds containing carboxylic acid. The ion exchange capacity (IEC) of these blends was amplified by doping these with β NSA and PhSA [7,12].

The influence of dopants on the synthesis and properties of PANI have been studied previously; however, to the best of our knowledge, the effects of β -naphthol sulfonic acid (β NSA) and phenol sulfonic acid (PhSA) on the synthesis and properties of conducting PANI have not been considered previously. The sulfonic group has more ionizing functionality, increasing the conductivity of PANI membranes and making the PANI surface more permeating for different types of ions for selective separation.

In electrodialysis, the separation of ionic substances takes place through ion exchange membranes by applying the electric field as a driving force. These membranes have their applications in the electrolytic cell, where they allow the selective transport of anions and cations [13]. Properties of the transport of ions depend on the nature of the polymer matrix, extent of the crosslinking, and fixed concentration of the ionic charges [14]. These carboxylic acid-containing compounds are the fermentation product of sugar molasses and starch hydrolysates [15,16]. Subsequently, it is essential to remove these from the fermented components. The extraction and precipitation of these organic acids requires a large amount of reagents and generate a substantial volume of waste water containing a higher concentration of mineral salts. Therefore, such electromembranes are not only efficient in the separation and purification of carboxylic acid-containing compounds from the fermented broth, they also make the process environmentally benign [16].

## 2. Experimental

### 2.1. Chemicals

Aniline, TPU (commercial grade), and hydrochloric acid (HCl) (Merck, Darmstadt, Germany), ammonium persulfate and glycine-hydrochloride 98% (Sigma-Aldrich, St. Louis, MO, USA), ammonia (BDH Ltd., Dorset, UK), β NSA, and PhSA were obtained from a local supplier and used as received. All reagents were prepared in deionized water.

### 2.2. Apparatus

Scanning electron microscopy (SEM) photographs were obtained with a JEOL JSM-6480LV scanning electron microscope (Tokyo, Japan) at different magnification levels. The FTIR- attenuated total reflectance (ATR) analysis of the PANI membranes were recorded with a JASCO FTIR 4100 spectrometer (Tokyo, Japan) in ATR mode, and ranged from 650–4000 cm−1 . The electrical conductance of membranes was measured using the 4-probe method, using a Keithley 6220 precision current source and 2182 nanometer. Thermogravimetric analysis (TGA) of the membranes was performed at a heating rate of 20 °C min−1 under a nitrogen atmosphere using a Schimadzu TGA-50 analyzer (Kyoto, Japan).

### 2.3. Preparation of PANI and doping with β NSA and PhSA

Aniline was polymerized using a standard method [17, 18]. Next, the polymerized reaction mixture (doped PANI) was filtered and treated with 100 mL of 1.1M ammonia solution (dedoping) to convert the emeraldine salt into an emeraldine base (EB). Finally, the ammonia-treated mixture (EB) was also filtered and redopped again with 50 mL of 1.5 M of β NSA and 50 mL of PhSA, one-by-one, with constant stirring for 24 h. The resulting β NSA- and PhSA-doped PANI were filtered and dried in an oven at 50 °C.

### 2.4. Synthesis of TPU/PANI-doped membranes

**Figure 1:**
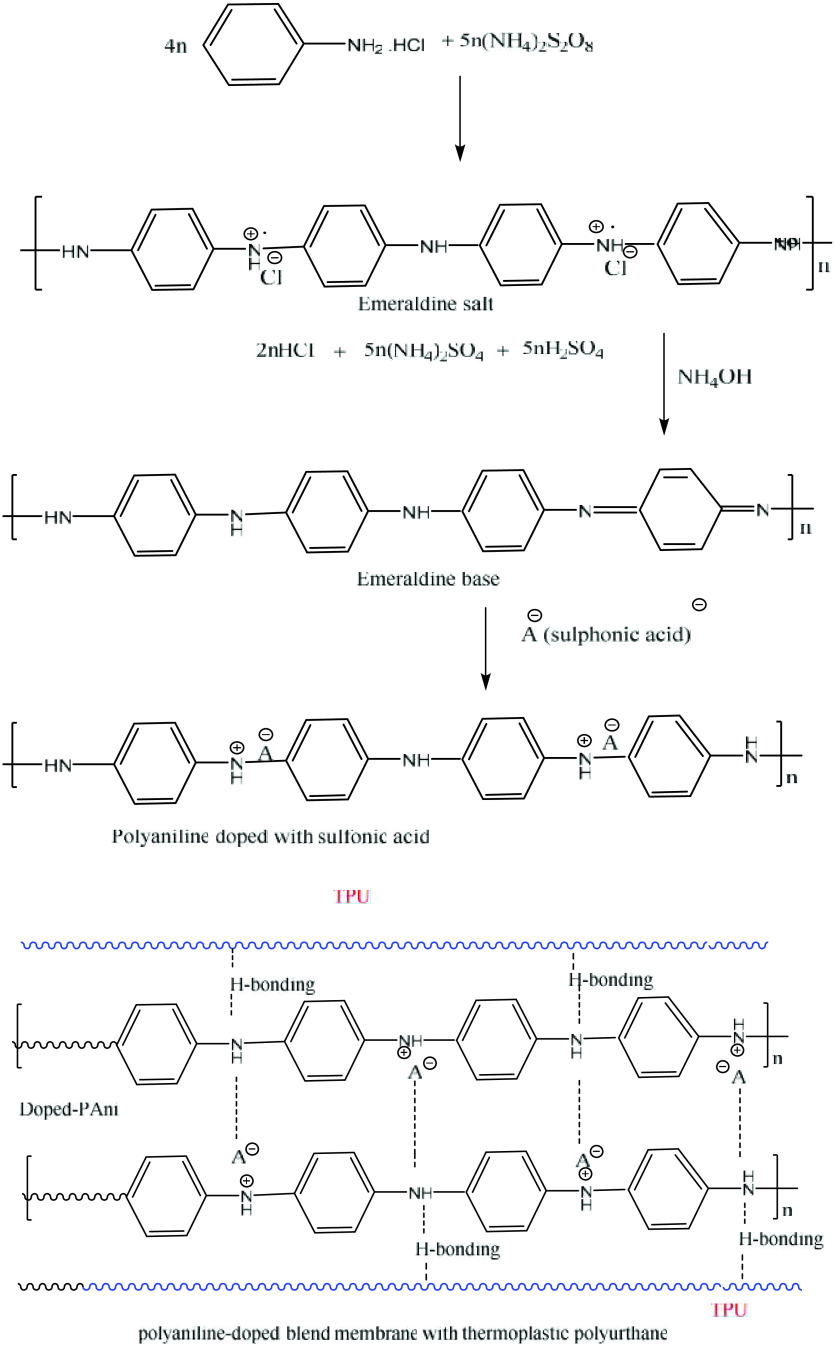
Schematic route for synthesis of the blended cation exchange membranes of the PANI.

Different composite PANI membranes were prepared by blending the β NSA- and PhSA-doped PANI and TPU at mixture ratios of 10%:90% and 20%:80%, separately. To achieve the ratio of 10%:90%, 0.4 g of PANI-sulfonic acid and 3.6 g of TPU were dissolved in 20 mL of dimethylforamide (DMF) solvent, and to achieve the ratio of 20%:80%, 0.8 g of PANI-sulfonic acid and 3.2 g of TPU were dissolved in DMF, and then stirred for 24 h at 25 °C. Finally, using these dissolved solutions, the membranes were cast onto glass plates, individually, using casting knife, and the solvents were allowed to evaporate for 24 h. Next, these membranes were peeled off of the glass plates and placed in a desiccator for further characterization. The scheme of the reaction for the synthesis of the PANI-blended cation exchange membranes is given in Figure 1.

### 2.5. Water swelling

For measurement of the percentage of water uptake, membranes measuring 2 cm2 were dipped in deionized water at room temperature for 24 h for equilibration. After removal of the excess water on these membranes using blotting paper, the membranes were weighed. They were dried in an oven at 80 °C and then weighed again. Finally, the percentage of water uptake was measured by calculating the weight difference between the wet and dry membranes using the following equation [19–21]:

Percentage of water uptake=Wwet-WdryWdryx100

### 2.6. Ion exchange capacity

A 2-cm2 sample of each membrane was first pretreated with 50 mL of IM HCl. Next, fully protonated TPU/PANI-doped membranes were submerged in 50 mL of 2M NaCl solution, separately, for 24 h, to exchange protons (H+) from the membranes with Na+ ions. The residual HCl solution was then titrated against 0.025M of NaOH solution using phenolphthalein as an end point indicator (colorless-light pink). The membrane specimens 226 were dried in oven at 80 °C to attain their dry weight (Wdry) . The IEC was determined using the following formula:

IEC(meq/g)=VNaOH.CNaOHWdry

### 2.7. Electrodialysis

**Figure 2:**
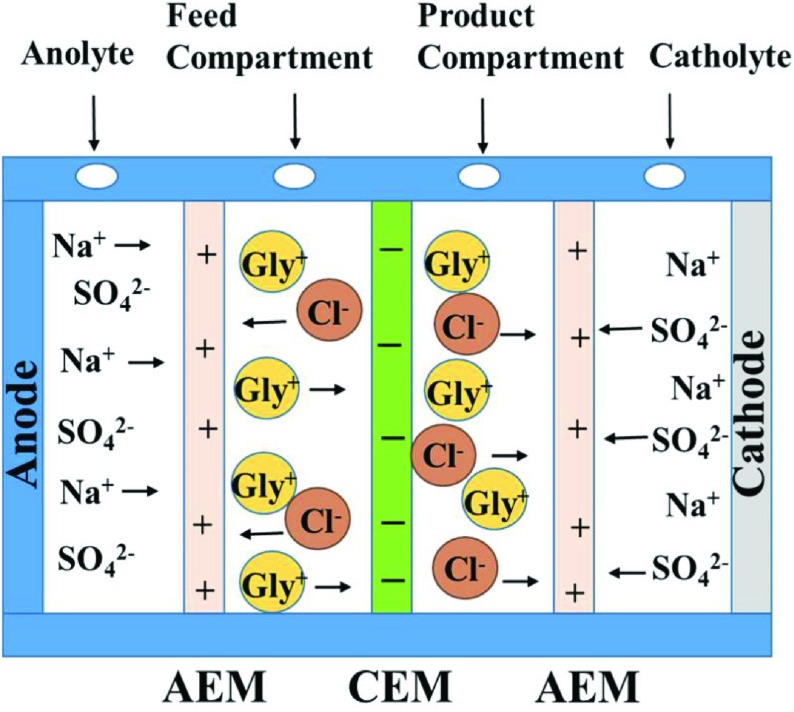
Experimental setup of the electro-dialyzer.

The electrodialysis process was performed in a 4-compartment cell, as shown in Figure 2. Titanium electrodes were used as the cathode and anode. The exposed area of these electrodes was 12.56 cm2 . The prepared PANI cation exchange membranes were used for separation of the glycine. The surface area of the membranes was 12.56 cm2 . The compartment alongside the anodic chamber was labeled the feed compartment and an anion exchange membrane was installed between these anodic and feed compartments. Next, 8.5 mL of 0.1N Na2 SO4 solution at pH 7.1 was added into the anodic compartment and 8.5 mL of 1% glycine hydrochloride solution at pH 3.5 was added into the feed compartment. The compartment beside the cathodic chamber was labeled the product compartment and an anion exchange membrane was installed between the cathodic and product compartments. Next, 8.5 mL of 0.1N Na2 SO4 solution at pH 7.1 was added into the cathodic compartment and 8.5 mL of 1% glycine hydrochloride solution at pH 3.5 was added into the product compartment. A cation exchange membrane was then installed between the feed and product compartments.

Before performing the electrodialysis operations, the membranes were immersed in their respective solutions for 24 h. All of the solutions were prepared with deionized water. The electrodialysis operations were performed at room temperature for 60 min at 30 V. The concentration of glycine was determined using the acid-base titration method with phenolphthalein as an indicator [22]

## 3. Results and discussion

### 3.1. Water swelling

Table 1 shows the results of the percentage of water uptake of the synthesized membranes. The TPU/10% PANI-β NSA absorbed 17.64% of the water because of the hydrophilic nature of the sulfonic group in the dopant acid used in the PANI membrane. The TPU/20% PANI-β NSA absorbed 29.03% of the water, and this significant increase was attributed to the higher contents in the sulfonic group. However, the percentages of water uptake in TPU/10% PANI-PhSA and TPU/20% PANI-PhSA were 24.54% and 35.25%, respectively. Water swelling of the membrane is directly correlated with the doping acid, where as the concentration of the doping acid is increased, the water swelling is also increased.

**Table 1:** Water uptake (%) values of pristine TPU and TPU/PANI-doped membranes.

Sr No.	PANI-doped membranes	Percentage of water uptake
1	TPU/10% PANI-βNSA	17.64
2	TPU/20% PANI-βNSA	29.03
3	TPU/10% PANI-PhSA	24.54
4	TPU/20% PANI-PhSA	35.25

The water uptake of PhSA-doped membrane was higher than that of the β NSA-doped membrane due to its structural interaction with the water. The structure of PhSA comprises a monoaromatic ring with hydroxyl and sulfonic functionality; thus resulting in increased water interaction due to hydrogen bonding. On the other hand, the structure of β NSA comprises a naphthalene ring with hydroxyl and sulfonic functionality, which has less hydrogen bonding and ultimately, less water uptake character in the membrane.

### 3.2. Ion exchange capacity

Table 2 represents the IEC values of various TPU/PANI doped with different sulfonic acid membranes. The IEC values were attributed to the exchangeable H+ of the TPU/PANI-doped membrane with the Na+ ion of the sodium chloride solution. The trend of the IEC resembled the percentage of water uptake. This was due to fact that, by increasing the percentage of doped PANI, the concentration of exchangeable H+ was also increased.

**Table 2:** IEC of TPU/PANI-doped cation exchange membranes.

Sr No.	Ion exchange membranes	Ion exchange capacity (meq/g)
1	TPU/10% PANI-βNSA	0.52
2	TPU/20% PANI-βNSA	0.78
3	TPU/10% PANI-PhSA	0.63
4	TPU/20% PANI-PhSA	0.97

### 3.3. Electrical conductivity

The results of electrical conductivity of the TPU-doped PANI are given in Table 3. The electrical conductivity of the TPU matrix was in the order of 10−14 Scm−1 [23] and it was increased by adding 10%–20% of the weight of PANI, up to 10−6 Scm−1 . In this study, the electrical conductivities of the prepared blends were in range of 1.57 ×10−6 to 4.78 ×10−6 Scm−1 . These values of the electrical conductivities were comparable with the literature established by other researchers [1, 24–26]. The results of the electrical conductivities led to the conclusion that PANI form a conductive-phase by intermixing with the TPU matrix, which might have resulted in enhanced electrical conductivity with loading of the higher contents of PANI in TPU [27]. It has also been reported that stronger H-bonding between PANI and TPU blend resulted in better miscibility and responsible to higher electrical conductivity [28].

**Table 3:** Electrical conductivities of TPU/PANI-doped cation exchange membranes.

Ion exchange membranes	Conductivity (S/cm)
TPU/10% PANI-βNSA	1.57 ×10−6
TPU/20% PANI-βNSA	3.38 ×10−6
TPU/10% PANI-PhSA	2.68 ×10−6
TPU/20% PANI-PhSA	4.78 ×10−6

### 3.4. Infrared spectroscopy

**Figure 3:**
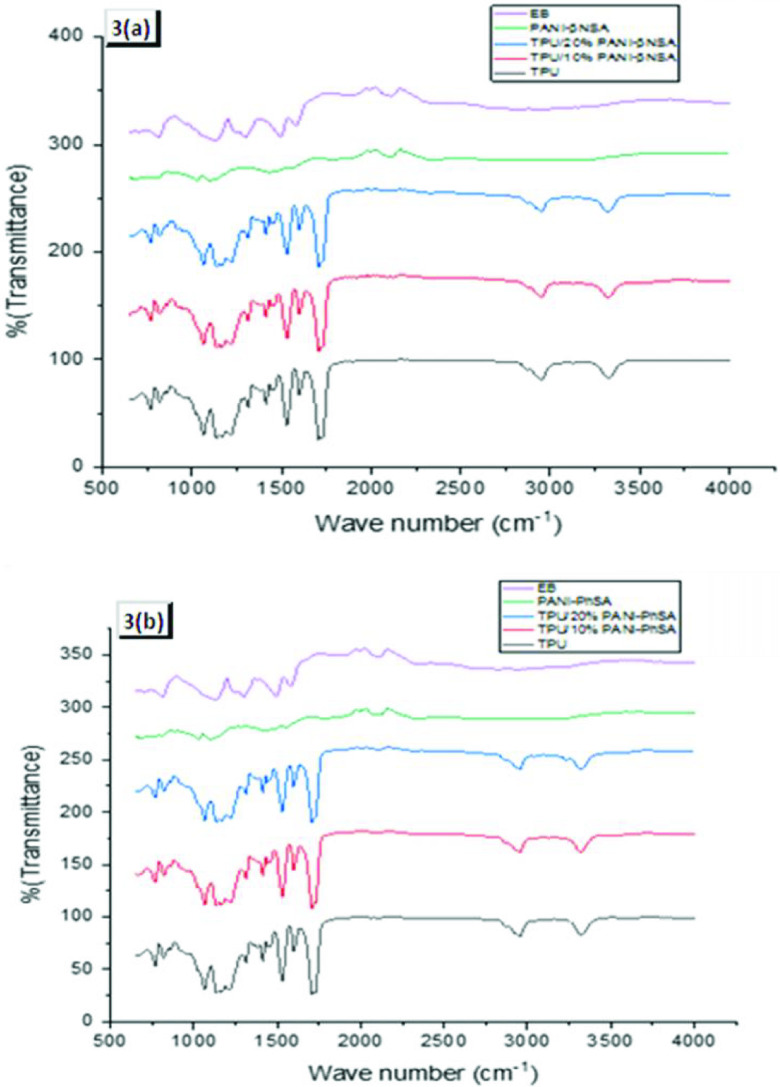
Comparative FTIR spectra of the membranes. (a) Spectra of pure TPU, TPU/10% PANI-β NSA, TPU/20% PANI-β NSA, and EB. (b) Spectra of pure TPU, TPU/10% PANI-PhSA, TPU/20% PANI-PhSA, and EB.

Due to the similar structures of PANI and TPU, the resulting composite membranes were quite similar, although there was a minute difference of the doping agent. Hence, it could be clearly seen that the FTIR graph of the pure TPU, TPU/10% PANI-β NSA, TPU/20% PANI-β NSA, and emeraldine base (EB) is given in Figure 3a, while Figure 3b shows the spectra of the pure TPU, TPU/10% PANI-PhSA, TPU/20% PANI-PhSA, and EB. The peaks, appearing at 1528 cm−1 and 1416 cm−1 , were attributed to quinoid and benzenoid ring deformation of the EB. PANI doping can also be seen at 1133 cm−1 , due to the formation of polaron H+N=Q=NH+. The peak observed at 1305 cm−1 shows the C-N stretching of secondary aromatic amine and the peak observed at 829 cm−1 shows the C-H out-of-plane bending mode. The peak at 1063 cm−1 was attributed to the S=O group, showing the association with the sulfonic acid used as the dopant. In the spectrum of polyurethane, various peaks are seen. The peak appearing at 2952 cm−1 shows the existence of the C-H stretch band. Peaks at 1707 cm−1 and 1598 cm−1 correspond to the free and H-bonded stretching vibration of the carbonyl (C=O) group. The peak at 1063 cm−1 was attributed to the C–O–C group [29] and incorporation of the doped PANI in TPU was evident by the shifting of the N-H stretch band at 3323 cm−1 in pure TPU, to lower the wave number, i.e. 3320 cm−1 and 3321 cm−1 , for the 90%:10% and 80%:20% TPU-doped PANI blends, respectively. In the spectrum of the membrane, the peaks of PANI and the TPU were observed, showing the incorporation of PANI into the TPU.

### 3.5. Thermogravimetric analysis

**Figure 4:**
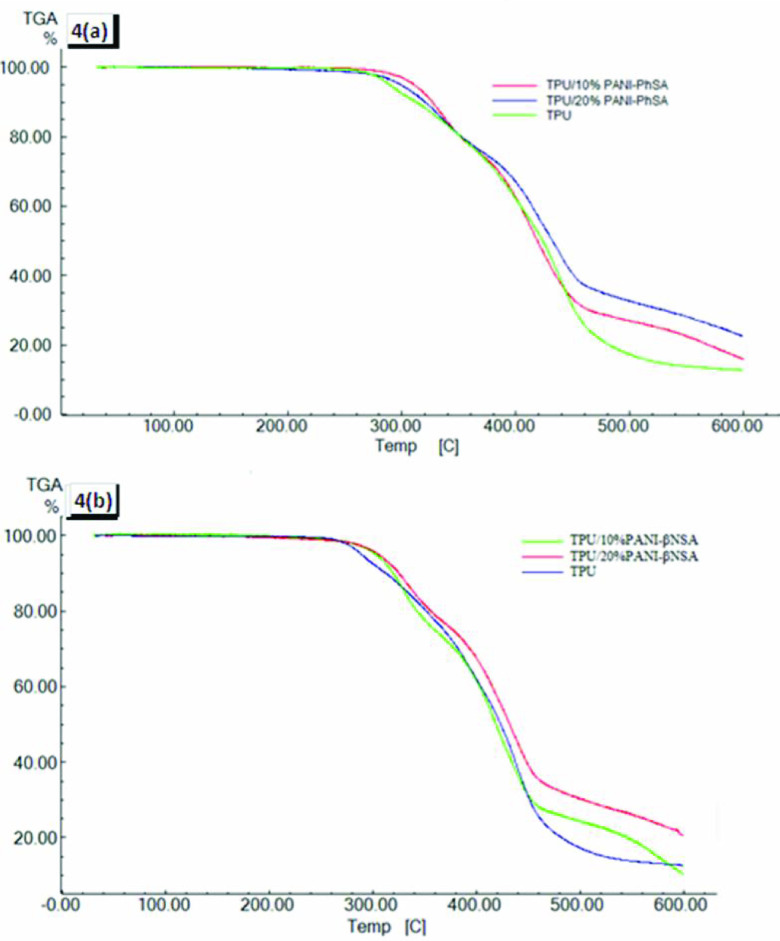
Thermogravimetric analysis. (a) Pure TPU, TPU/10% PANI-PhSA, and TPU/20% PANI-PhSA. (b) Pure TPU, TPU/10% PANI-β NSA, and TPU/20% PANI-β NSA.

TGA graphs of pure TPU and its blends with doped PANI representing 2 steps of degradation are shown in Figures 4a and 4b. The first step of degradation corresponded to the PANI hard segment and toluene diisocyanate (TDI) (hard segment of TPU), while the second step of degradation was attributed to polyols (soft segment of polyurethane). It was observed from the TGA curves that by adding the doped PANI into the TPU matrix, the thermal stability of the blends was also enhanced. For pure TPU, the initial weight loss was observed at 280 °C to 425 °C and the second was at 425 °C to 460 °C. The addition of 10% and 20% doped PANI increased the first and second degradation temperatures up to 22 °C and 25 °C, respectively. The amine group of PANI formed urea linkage with isocyanate moiety of the TDI and contributed as the hard part of TPU. Hence, by increasing the doped PANI contents in the TPU matrix, the number of urea linkages was also increased, which were more thermally stable than the urethane linkages. These urea linkages were responsible for the enhanced stability of these blends. The maximum rate of decomposition of pure TPU was 430 °C and 460 °C. Moreover, it was also seen that by adding the PANI contents into the TPU matrix, char residue was increased. In many studies, it was observed that char residues resulted in the thermal stability of the blends [30, 31], showing that the aromatic rings of PANI were not fully decomposed, even at high temperatures in the inert atmosphere of nitrogen [32].

### 3.6. SEM images

**Figure 5:**
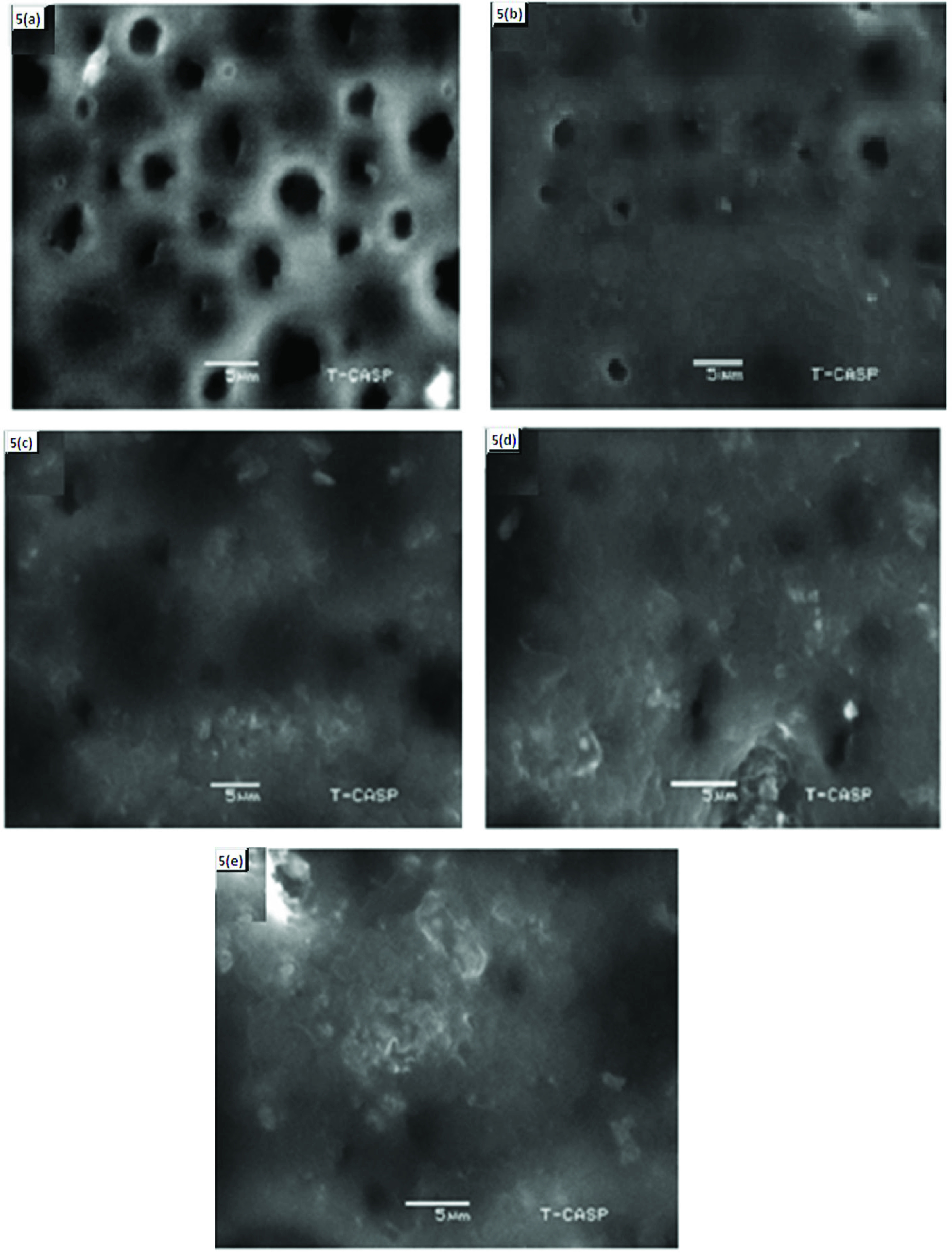
SEM images of the membranes. (a) Pure (TPU) membrane. (b) Images of TPU/10% PANI-PhSA, (c) TPU/10% PANI-β NSA, (d) TPU/20% PANI-PhSA, and (e) TPU/20% PANI-β NSA.

Figure 5 represents the SEM image of pure TPU membrane and different ratios of doped PANI and TPU. Figure 5a shows the SEM image of pure TPU, representing its porous structure. Figures 5b and 5c, show that by adding 10% PANI-PhSA and 10% PANI-β NSA into the TPU matrix, most of the pores interacted with the doped PANI via Van der Waals force, while Figures 5d and 5e, show that increasing the doped PANI-PhSA and PANI-β NSA ratio up to 20% reduces the porous structure of TPU and converts it into a dense membrane, which changes the apparent morphology of the membrane. Ion transport was affected by the morphological differences of these matrixes. Dense membrane allows the passage of ions through water in the hydration layer, and provides greater mechanical strength than a porous one [33, 34].

### 3.7. Electrodialysis

**Figure 6:**
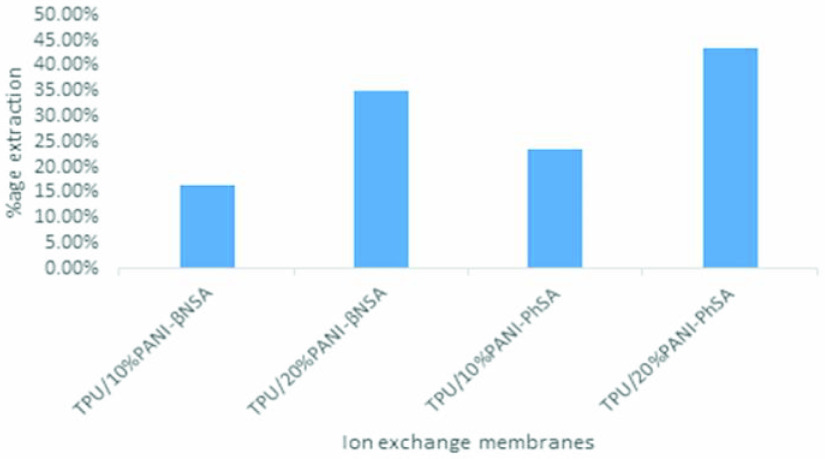
Percentage extraction of glycine using electrodialysis by TPU/10% PANI-PhSA, TPU/20% PANI-PhSA, TPU/10% PANI-β NSA, and TPU/20% PANI-β NSA ion exchange membranes.

Figure 6 and Table 4 show the percentage of glycine extraction for TPU/10% PANI-β NSA, TPU/20% PANI-β NSA, TPU/10% PANI-PhSA, and TPU/20% PANI-PhSA. The percentage of glycine extraction increased by increasing the amount of PANI from 10%–20% due to an increased concentration of the sulfonic groups. The types of acids that were used as the PANI dopant had less influence on transport, in a range of concentrations up to 10%, because the dopant acids had different structures and they represented interaction with the studied ions.

**Table 4:** Percentage extraction of glycine by different membranes of TPU/PANI-doped with sulfonic acid for electrodialysis during a 60-min time frame.

Sr No.	Ion exchange membranes	Initial glycine concentration in the feed compartment (g/100 mL)	Final glycine concentration after electrodialysis in the feed compartment (g/100 mL)	Difference between the initial and final glycine concentrations (g)	Percentage of glycine extraction by electrodialysis (%)
2	TPU/10% PANI-βNSA	1.020	0.851	0.169	16.5%
3	TPU/20% PANI-βNSA	1.020	0.662	0.358	35.09%
4	TPU/10% PANI-PhSA	1.020	0.778	0.242	23.7%
5	TPU/20% PANI-PhSA	1.020	0.578	0.442	43.33%

### 3.8. Conclusions

Cation exchange membranes were prepared by blending TPU with different concentrations of PANI doped with various sulfonic acids (PANI-sulfonic acid). SEM images of these TPU/PANI-sulfonic acid membranes showed that as the percentage of PANI increased, the porous matrix of TPU tended to change into a dense structure. The identification of particular interaction between TPU and PANI was verified by FTIR analysis, while the TGA analysis showed that the thermal stability of these membranes increased by increasing the ratio of PANI to sulfonic acid. It demonstrated that degradation of the TPU/PANI-sulfonic acid membrane started at 280 °C. Hence, these membranes can be used in separation processes below this temperature. The water uptake, IEC, electrical conductivity (via the 4-probe method), and glycine separation efficiency (by electrodialysis) values increased by increasing the percentage of PANI-sulfonic acid contents into the TPU. It was observed that due to the more hydrophilic nature of PhSA, it exhibited more positive results than β NSA as a dopant. These novelly developed membranes can be used to separate the effluent load of feed industries that have higher contents of amino acids.
